# Strain Development in Microalgal Biotechnology—Random Mutagenesis Techniques

**DOI:** 10.3390/life12070961

**Published:** 2022-06-27

**Authors:** Richard Bleisch, Leander Freitag, Yob Ihadjadene, Una Sprenger, Juliane Steingröwer, Thomas Walther, Felix Krujatz

**Affiliations:** 1 Institute of Natural Materials Technology, Technische Universität Dresden, 01069 Dresden, Germany; richard.bleisch@tu-dresden.de (R.B.); leander.freitag@posteo.de (L.F.); yob.ihadjadene@tu-dresden.de (Y.I.); una.sprenger@posteo.de (U.S.); juliane.steingroewer@tu-dresden.de (J.S.); thomas_walther@tu-dresden.de (T.W.); 2 Biotopa gGmbH—Center for Applied Aquaculture & Bioeconomy, 01454 Radeberg, Germany; 3 Faculty of Natural and Environmental Sciences, University of Applied Sciences Zittau/Görlitz, 02763 Zittau, Germany

**Keywords:** random mutagenesis, algae, mutagens, strain development, microalgal biotechnology

## Abstract

Microalgal biomass and metabolites can be used as a renewable source of nutrition, pharmaceuticals and energy to maintain or improve the quality of human life. Microalgae’s high volumetric productivity and low impact on the environment make them a promising raw material in terms of both ecology and economics. To optimize biotechnological processes with microalgae, improving the productivity and robustness of the cell factories is a major step towards economically viable bioprocesses. This review provides an overview of random mutagenesis techniques that are applied to microalgal cell factories, with a particular focus on physical and chemical mutagens, mutagenesis conditions and mutant characteristics.

## 1. Introduction

Microalgae comprise various pheno- and genotypes of eukaryotic (microalgae) and prokaryotic bacteria (cyanobacteria). They typically live in freshwater, soil or marine habitats but also more extreme habitats, such as salt, sulfur-rich lakes or on snow surfaces [1]. These organisms are able to produce a wide range of industrially relevant products, such as carotenoids (astaxanthin, β-carotene, lutein) [2,3,4], pigments (phycobiliproteins, e.g., phycocyanin) [5], polysaccharides (hydro colloids, e.g., sulphated polysaccharides) [6], vitamins (vitamin B12) [7] and starch [8]. They can assemble various lipids, including polyunsaturated and omega-3 fatty acids (e.g., eicosapentaenoic acid or docosahexaenoic acid) [9,10], trans-fatty acids [11] and fatty acid methyl esters [12,13].

There are two major approaches to improve the performance of cell factories: rational metabolic engineering or random mutagenesis [14]. Rational metabolic engineering aims to optimize metabolic pathways by the targeted manipulation of enzymatic activities, i.e., involving knock-out, overexpression or new enzymatic conversions in the cell. The usage of the metabolic toolboxes requires a systematic knowledge of the metabolism and its regulation inside the microbial cell factory, supported by genome-based methods, such as next-generation sequencing [15], proteomics [16] and metabolomics [17,18,19,20]. However, there are still regulatory issues related to the usage of genetically modified organisms in industrial fields, such as the food and feed industry or natural cosmetics. As a consequence, alternative nature-based strategies must be applied to obtain advanced cellular factories.

The concept of random mutagenesis involves an iterative exposure to physical or chemical mutagens, yielding a genetic and phenotypic diversity of mutants, which have to be screened for the desired cell properties and improved metabolic functions [18,19,20]. In this review, a broad overview of technologies for inducing random mutations in microalgae and cyanobacteria is presented. It should be noted that the specific effects at the genetic level are not yet known for each mutagen. The core of this review is formed by the tabular overviews, in which recent studies on random mutagenesis are presented, focusing on the methodology and the results obtained. For a detailed description of the methods, please refer to the respective literature sources.

## 2. Mutagens Applied to Microalgae for Random Mutagenesis

**Figure 1:**
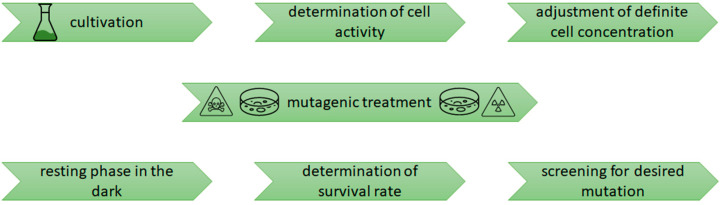
Workflow of random mutagenesis process applied to microalgae.

The success of a random mutagenesis approach using microalgae is determined by multiple factors involving the treatment of the cells before, during and after the mutagenesis procedure (Figure 1). Using photosynthetic microalgae, the supply of light quality and quantity [21,22], as well as the supply of carbon and nitrogen, are the most important factors [21,22,23,24]. Besides the environmental conditions, the type of mutagen, its concentration and exposure time are among the main factors affecting the mutation result.

**Figure 2:**
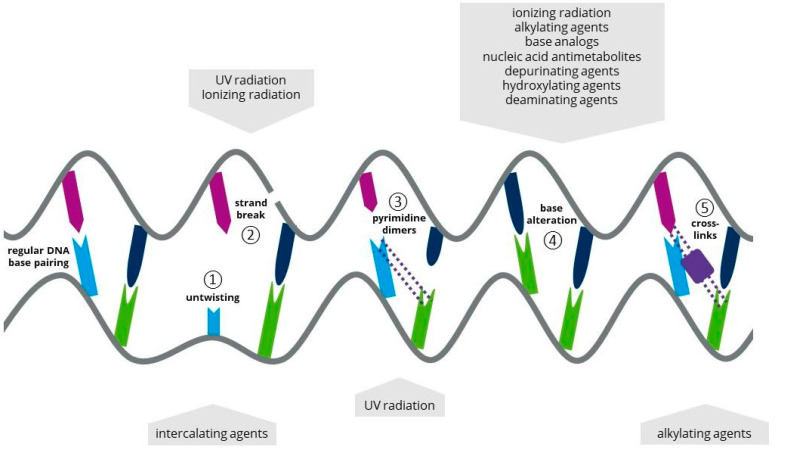
Mutagens and their impact on DNA. Five different alterations in DNA are shown: (**1**) DNA strands are untwisted by intercalating agents (chemical mutagen). (**2**) A single- or double-strand break is induced by UV radiation or ionizing radiation (physical mutagens). (**3**) Pyrimidine dimers, covalent binding between two pyrimidine bases, are introduced by UV radiation (physical mutagen). (**4**) Different chemical mutagens can cause base alterations in DNA. (**5**) Cross-links are formed by alkylating agents (chemical mutagen).

A mutagen leads to irreversible changes in the cell’s genetic information [25,26,27] with the goal to create vital mutant cells with great genetic and phenotypic variety. Figure 2 presents an overview of alterations to the deoxyribonucleic acid (DNA) potentially induced by several types of mutagens.

In order to evaluate the quality of a mutagenesis event, several parameters can be monitored, such as the cell’s survival rate or the mutation rate. However, a general standardization of mutagenesis involving culture conditions and monitoring parameters is not available yet and is dependent on the experience of the scientists involved and the lab infrastructure. An important parameter to adjust a suitable mutagen concentration is given by the cell survival rate, representing the viable cells after the mutagenic treatment. For this purpose, cell-impermeant dyes are commonly used. They are unable to cross intact membranes and can, therefore, be used for the analysis of cell viability and membrane integrity, allowing the estimation of the percentage of dead and vital cells [28,29]. Several dyes, such as SYTOX Green [30,31], propidium iodide [32], methylene blue, trypan blue, eosin and nile blue [29], have been applied so far. Most studies aim for a survival rate of 5–20% to reach a good mutation rate within the surviving cell population [33,34,35].

After the exposure to the mutagen, almost all studies keep the cells in the dark for at least overnight or up to 24 h [36,37,38,39,40,41] in order to decrease the photoactivation of cellular repair pathways, such as the (6-4) photolyases.

While most studies using random mutagenesis do not quantify the resulting mutation rates, the spontaneous mutation rate for microalgae varies between 3.23 × 10^−10^ µ for *Chlamydomonas reinhardtii* [42] and 10.12 × 10^−10^ µ for *Picochlorum costavermella* [43], with µ as the mutation rate per nucleotide per generation. However, for varying mutagens, the mutation rate is higher, e.g., 1.4 × 10^−5^ for the chemical mutagen TNT in *Dictyosphaerium chlorelloides* [44].

### 2.1. Physical Mutagens in Microalgal Biotechnology

#### 2.1.1. Ultraviolet Light

UV radiation is mainly used to generate random mutations in microalgal cells. Depending on the wavelength, UV radiation is classified as UV-A (315–380 nm), UV-B (280–315 nm) and UV-C (200–280 nm). As presented in Figure 2, UV exposition induces several types of DNA alterations; however, it has to be taken into account that phototrophic cells might be resistant to certain physical mutagens due to their photon-capturing and quenching properties. For instance, *Zygmena circumcarinatum* and *Chlorella protothecoides* revealed a high resistance to ionizing radiation, while *Nostoc* sp., *Stylidium javanicum* and some extremophiles showed UV protective properties [45,46,47,48,49,50].

Further, 80% of mutation events caused by UV, especially UV-C radiation, are related to the formation of pyrimidine dimers within the DNA. 5-methylcytosine is frequently involved in this type of mutation as it deaminates spontaneously to thymine; hence, the energy absorption shifts to higher wavelengths compared to non-methylated cytosine. Additionally, pyrimidine(6-4)pyrimidone photoproducts can be formed by UV radiation with neighboring pyrimidines between positions 6 and 4 [51,52]. Radiation at 260 nm (UV-C) leads to the most efficient formation of cyclobutene pyrimidine dimers and 6-4-photoreaction products, as DNA absorption reaches its maximum level at this spectral range. Therefore, UV-C irradiation has been recommended for random mutagenesis approaches, including microalgae [53]. A comprehensive overview on UV-radiation-induced mutagenesis approaches is presented in Table 1.

#### 2.1.2. Ionizing Radiation

Ionizing Radiation, such as gamma irradiation, X-rays or ion beams, can also act as physical mutagens [54]. Due to the higher energy density compared to UV radiation, ionizing radiation causes serious genetic damages [55], such as the ionization of molecules, the alteration of bases, the breaking of phosphodiester bonds and the production of chromosomal aberrations, such as deletions, translocations and chromosomal fragmentation [56].

In view of the lack of knowledge on interactions between gamma radiation and microalgae, Gomes et al. [57], investigated the effects of various gamma ray intensities on the green alga *Chlamydomonas reinhardtii*, revealing modifications to the PSII energy transfer and a decrease in photosynthetic activity due to the induced formation of reactive oxygen species (ROS) by gamma radiation. Senthamilselvi and Kalaiselvi [58], analyzed the effects of gamma radiation on the microalgae *Chlorella* sp. in a range of 100 Gy to 1100 Gy, showing a 1.4-fold increase in the intracellular neutral lipid content compared to the wild type. Even the biomass production increased in 10 out of 12 mutants compared to the wild type by up to 27.16%.

#### 2.1.3. Atmospheric and Room Temperature Plasma

New physical mutagenesis approaches have been recently presented using atmospheric and room temperature plasma (ARTP) for several bacterial and microalgal strains [59]. ARTP approaches involve the exposition of cells to charged particles [60], electromagnetic fields [61], neutral reactive species [62] and heat [63]. Due to low, controllable gas temperatures, the rapid performance, the high diversity of mutants and the tool’s environmentally friendly operation, ARTP mutagenesis shows high potential; however [64], comprehensive datasets, including survival rates of cells or the mutation rate, are not available yet [59].

#### 2.1.4. Laser Radiation

The use of laser radiation in the near infrared and visible spectrum has already been reported for fungi and bacteria [65]. In recent years, it has also been adapted for microalgae. Due to natural heat dissipation and fluorescence quenching, many microalgae show a higher tolerance to radiation in the visible light spectrum. For a significant mutagenesis effect, higher intensity has been realized by using lasers, including semiconductor lasers (632.8 nm), (He-Ne) lasers (808 nm) or Nd:YAG lasers (1064 nm). This mutagenesis approach provides short-term exposure of microalgae in the minute range. Due to the ease of application and the good results obtained in initial studies, e.g., for the improvement in lipid production, there still seems to be potential [66,67]. Table 1 provides an overview on physical mutagens applied to microalgae.

**Table 1:** Physical mutagens applied to microalgae.

Mutagen	Method, Exposure Time, Source, Distance, Recovery Time	Reference Microalgae	Mutation Results			References
			Mutated trait	WT *	M **	
UV	UV 18 W, for 13 min, 15 cm, 24 h darkness	*Chlorella vulgaris* Y-019	neutral lipid accumulation [g/g dry wt]	0.11	0.26	[36]
UV-C	UV-C 253.7 nm, 30-W, 3–30 min, 9 cm, 24 h darkness	*Chlorella* sp.	protein content [g/L]	0.0242	0.0688	[37]
	UV-C 254 nm 1.4 mW/cm^2^ for 60 s, 15 cm, 16 h darkness	*Chlorella vulgaris*	fatty acids 16:0;18:0, 20:0 [% of total fatty acids]	27.9; 3.9; 11.9	47.4; 5.9; 19.9	[68]
	UV-C 254 nm, 15 W, (Vilber–Lourmat, France), for 30–180 s, 5 cm, 24 h darkness	natural isolates of photosynthetic microorganism	lipid content though Nile red autofluorescence; with fluorescence emission	35; 1081	983; 89,770	[38]
	UV-C 40,000 μJ/cm, 254 nm, overnight darkness	*Scenedesmus obliquus*	trans-fatty acid productivity [g/(L·d)]	0.095	0.112	[69]
	UV-C 254 nm 340 mW cm^2^, for 3–32 min, 13.5 cm, 24 h darkness	*Isochrysis affinis galbana*	total fatty acid [g/g dry wt]	0.262	0.409	[40]
	UV-C, for 1–10 min, 40 cm, overnight darkness	*Chlorella vulgaris*	lipid content [g/g]	0.58	0.75	[35]
Gamma irradiation	10 doses of irradiation 50–7000 kGy, 60Co gamma ray irradiator, room temperature	*Scenedesmus* sp.	lipid productivity [g/L·d]	0.0648	0.097	[70]
ARTP	He RF power 100 W, plasma temperature 25–35 °C, for 20; 40; 60 and 80 s, 2 mm	*Spirulina platensis*	Carbohydrates productivity [g/L·d]	0.0157	0.026	[59]
	He RF power 100 W, plasma temperature 25–35 °C, 20–60 s, 2 mm	*Chlamydomonas reinhardtii*	H_2_ production [mL/L]	~16.1	84.1	[71]
	He RF power 150 W, for 100 s	*Crypthecodinium cohnii*	biomass concentration [g dry wt/L]	3.60	4.24	[72]
Heavy ion beam	^12^ C^6+^ ion beam 31 keVµm^−1^ 160 Gy,	*Nannochloropsis oceanica*	lipid productivity [g/L·d]	0.211	0.295	[73]
	^12^ C^6+^ ion beam, 90 Gy	*Desmodesmus sp.*	lipid productivity [g/L·d]	0.247	0.298	[74]
Low-energy ion beam implementation	N+ ion beam chamber pressure 10^−2^ Pa Dose of implantation 0.3–3.3·10^15^ ions cm^−2^ s^−1^	*Chlorella pyrenoidosa*	lipid productivity [g/ L·d]; Lipid content [g/g dry wt]	47.7; 0.337	64.4; 0.446	[75]
laser radiation	He–Ne laser 808 nm, 6 W, 4 min, 24 h darkness	*C. pyrenoidesa*	lipid content [g/g dry wt]	0.354	0.780	[66]
	Nd:YAG laser 1064 nm, 40 mW 8 min, 24 h darkness	*Chlorella vulgaris*	lipid content [g/g dry wt]	0.315	0.525	[66]
	Nd:YAG laser 1064 nm, 40 mW 2 min, 24 h darkness	*Chlorella pacifica*	lipid content [g L^−1^]	0.033	0.088	[76]
	semiconductor laser 632 nm, 40 mW, 4 min, 24 h darkness	*Chlorella pacifica*	lipid content [g L^−1^]	0.033	0.077	[76]

* Wildtype, ** Mutant.

### 2.2. Chemical Mutagens in Microalgal Biotechnology

#### 2.2.1. Alkylating Agents as a Chemical Mutagen

Alkylating agents (AAs) are commonly used in random mutagenesis to induce nucleotide substitutions within the DNA. AAs transfer alkyl residues, predominantly methyl and ethyl groups, yielding a change in base pairing, followed by typical point mutations after replication of the DNA. It was observed that chloroethylating drugs can also cause sister chromatid exchange or DNA breaks [77], even though AAs cannot induce the direct scission of the DNA backbone [78]. Alkylation leads to the formation of adducts on either O- or N-atoms of nucleotides or O-atoms in phosphodiesters. O-alkylations are particularly potent mutagens, while N-alkylations act predominantly cytotoxic rather than mutagenic [77,79].

One widely used chemical mutagen is ethyl methanesulfonate (EMS), which induces point mutations, in particular, by guanine alkylation, yielding an A·T→G·C transition. Other AAs (shown in Table 2) applied to induce random mutations include methylnitronitrosoguanidine (MNNG) [80], diethyl sulfate (DES) [81], N-methyl-N-nitrosourea (NMU) [82] or N-methyl-N′-nitro-nitrosoguanidine (MNNG) [83,84], which can methylate almost all O- and N-atoms, up to several hundred times more effectively than similar concentrations of other monofunctional AAs [78].

AAs have also been used in combination with other mutation approaches, such as exposure to UV radiation (MNNG and EMS) [85,86] or base analogs (MNNG) [78], in order to achieve a higher mutation rate.

#### 2.2.2. Base Analogs (BAs) as a Chemical Mutagen

Chemicals that are capable of replacing DNA bases during the replication process are called base analogs (BA). If the BA is chemically bound to deoxyribose, there is a possibility that it will change shape and, thus, pair with an incorrect base during replication. Depending on the BA used, different types of changes in DNA pairing can be induced [26,87].

5-bromodeoxyuridine (5BrdU) is a uridine/thymidine analog. If 5BrdU is bound to deoxyribose, it is capable of a tautomeric shift to its enol form, leading to a guanine–cytosine-base pairing after DNA replication (A·T → G·C) [33]. Since it changes the structure by tautomeric probability, it can also cause a mutation in the opposite way, pairing with thymine instead of cytosine (G·C → A·T) [88].

2-aminopurine (2AP) is an adenine analog that causes similar changes in DNA pairing to 5BrdU [89]. 5-azacytidine (5AZ) is one of the most commonly used cytidine analogs due to its unique mutagenic specificity, changing only from cytidine BA to a guanine BA (C·G → G·C) [90].

When combined, some BAs have been detected to show a higher mutagenic effect than they could normally accomplish on their own. Combining 2AP and zebularine (ZEB) resulted in a 35-fold increase in mutation frequency in *E. coli* [91]. Similar effects can be observed for the combination of BAs with other physical or chemical mutagens, such as UV radiation and AAs. The repair mechanisms activated by the mutagens increase the probability of the BAs being introduced into the DNA [26]. Similar mechanisms can be assumed using BAs to induce random mutations to microalgae [90]; however, further research is necessary in this field.

#### 2.2.3. Antimetabolites (AMs) as a Chemical Mutagen

The structure of AMs is very similar to metabolites that appear naturally in the cell, but they cannot fulfill their function. AMs, such as 5′fluoro-deoxyuridine (5′FDU) or 2-Desoxy-D-glucose, are inhibiting essential enzymes or mechanisms necessary for DNA replication [27,92]. AMs tend to have multiple mutating and cytotoxic effects, e.g., the pyrimidine analog 5′FDU. After biotransformation, 5′FDU inhibits the enzymatic transformation of cytosine nucleosides into their deoxy derivative and the incorporation of thymidine nucleotides into the DNA strand [92].

AMs have been successfully used as chemical mutagens for many bacteria and fungi species [27,92,93]. In combination with a physical mutagen, such as UV light, good mutagenesis results have been reported in recent studies [27]. However, applying AMs to microalgal cells is a future field of research.

#### 2.2.4. Intercalating Agents (IAs) as a Chemical Mutagen

IAs wedge between the DNA base pairs due to their particular shape. Streisinger et al. [94] recognized that this interaction often occurs in regions with repeated base pairs (e.g., CCCCC) during DNA replication. The bonds are reversible and non-covalent.

This intercalating leads to the deformation of base pairs, resulting in the untwisting and lengthening of the DNA strands. These structural modifications to the DNA affect many functions, such as transcription, replication and repair mechanisms, and may inhibit them or be mutagenic [95].

Acridine and its derivatives are the most widely used and studied DNA IAs. IAs can be mono-intercalators, bis-intercalators or both (such as echinomycin), often depending upon the length of the alkyl chain separating the chromophores [96,97].

Mono-intercalators appear either as frameshift mutations in bacteria or as non-mutagens. Bis-intercalators act as “petite” mutagens, e.g., in *Saccharomyces cerevisiae*, suggesting that they may be more likely to target mitochondrial than nuclear DNA. IA often introduces frameshifting mutations, which they are commonly used for [95]. Petite mutants are described by Ephrussi [98], as cells having defective or altered mitochondrial DNA, resulting in very small (“petite”) colonies [99]. In microalgae and other eukaryotes, IAs seem to introduce mutations, especially in the mitochondrial genome [97,100].

Most IAs, such as echinomycin and acridine and its derivatives, have so far mainly been studied for bacteria, bacteriophage and yeast. A wider use for the random mutagenesis of microalgae is still pending.

#### 2.2.5. Other Approaches for Chemical Mutagenesis

A vast number of other chemicals are described in fundamental biology literature [51,56], for example, deaminating agents (e.g., nitrite) or hydroxylating agents (e.g., hydroxylamine), which replace the amino group of bases with a hydroxyl group and cause alterations in base pairing. Cross-linking agents (e.g., psoralen) or adduct-forming agents (e.g., acetaldehyde) bind covalently to DNA bases and, thus, complicate DNA replication. Other chemical mutagens include mycotoxins (e.g., aflatoxin B1), which can cause indirect damage to metabolites [51,56]. Table 2 provides an overview of chemical mutagens applied to microalgae, their utilization and related results.

**Table 2:** Chemical mutagens applied on microalgae. * Derived from original data.

Mutagen	Mutagen Concentration, Time of Exposure	Reference Microalgae	Mutation Results			References
			Mutated trait	WT *	M **	
EMS	EMS 0.1–1.2 M for 60 min	*Nannochloropsis* sp.	fatty acid methyl esters [g/g of dry wt]	0.123	0.238	[101]
	EMS 0.4–1 g/L for 60–120 min	*Haematococcus pluvialis*	total carotenoid; Astaxanthin [g/g of dry wt]	0.02; 0.005	0.02; 0.019	[102]
	EMS 300 mM for 60 min	*Chlorella vulgaris*	protein content [g/g of dry wt]	0.353	0.455	[34]
	EMS 0.2–0.4 M for 2 h in darkness	*Chlorella vulgaris*	violaxanthin [mg/L culture]	1.64	5.23	[103]
	EMS 0.1–0.2 M	*Phaeodactylum tricornutum*	total carotenoids [g/g dry wt]	0.009	0.011	[104]
	EMS 0.2 M for 2 h in the dark	*Dunaliella tertiolecta*	Zeaxanthin [μg/10^6^·cells]	0.131	0.359	[105]
	EMS 20–40 µL/mL for 2 h	*Chlamydomonas* *reinhardtii*	fatty acid methyl esters yield [%]	6.53	7.56	[106]
	EMS 0.2 M for 2 h in the dark	*Dunaliella* *salina*	carotenoid synthesis [Mol Car/Mol Chl]	0.99	1.24	[107]
	EMS 100 μ mol mL^−1^, for 30 min	*Chlorella* sp.	lipid content [g/g of dry wt]; productivity [g/(L·d)]	0.247; 0.1536	0.356; 0.2487	[108]
	EMS 0.4 M, for 60 min	*Coelastrum* sp.	Astaxanthin content [g/L]	0.0145	0.0283	[109]
EMS + UV	UV + EMS 25 mM for 60 min	*Chlorella vulgaris*	lipid content [%]	100	167	[85]
	UV 5–240 s, 245 nm + EMS 0.24 mol/L for 30 min	*Nannochloropsis salina*	fatty acid methyl ester [g/g of dry wt]	0.175	0.787	[110]
MNNG	MNNG 0.1 mM for 60 min	*Haematococcus pluvialis*	Total carotenoid content [g/L]	~0.067	0.089	[80]
	MNNG 5 µg/mL for 60 min	*Chlorella* sp.	max. growth rate under alkaline conditions [ d^−1^]	0.064	0.554	[111]
	MNNG 0.02 mol/L for 60 min	*Nannochloropsis* *oceanica*	Total lipid content [g/g] Lipid productivity [g/(L·d)]	0.241; 0.0065	0.299; 0.0086	[33]
	MNNG 0.1–0.2 M	*Phaeodactylum tricornutum*	total carotenoids [g/g dry wt]	0.009	0.011	[104]
	MNNG 0.2 mg/mL	*Chlorella sorokiniana*	Lutein content [g/L]	0.025	0.042	[83]
	MNNG 0.25–0.5 mM	*Botryosphaerella* sp.	lipid [g dry wt/(m^2^ day)]; biomass productivity [g dry wt/(m^2^·day)]	1.0; 3.2	1.9; 5.4	[84]
NMU	NMU 5 mM for 60–90 min	*Nannochloropsis oculata*	Total fatty acid [g/g dry wt]	0.0634	0.0762	[82]
DES + UV	UV 7–11 min 254 nm + DES 0.1–1.5% (V/V) 40 min	*Haematococcus pluvialis*	astaxanthin content [mg/L]	~0.031	~0.089	[81]
5BU	5BU 1 mM for 48 h	*Chlamydomonas reinhardtii*	O_2_ tolerance [%]	100	1400	[112]
5′FDU	5′FDU 0.25 and 0.50 mM for 1 week	*Chlorella vulgaris*	fatty acids 16:0; 18:0; 20:0 [% of total fatty acids]	27.9; 3.9; 11.9	46.9; 5.5; 18.5	[68]
Acriflavin	Acriflavin 2–8 μg/mL for 1–3 d in darkness	*Chlamydomonas reinhardtii zyklo*	Loss of respiratory rate [nmol O_2_/(min·10^7^ cells)] through loss of mitochondrial DNA	23.2	3.7	[100]

* Wildtype, ** Mutant.

## 3. Further Approaches in Random Mutagenesis

Recently, combined mutagenesis approaches have generated high interest as results indicated that they have a higher success rate than individual approaches. For instance, Wang et al. [81] applied a two-step random mutagenesis protocol to *Haematococcus pluvialis* cells using first UV irradiation, then EMS and DES mutagenesis, causing astaxanthin production to increase by a factor of 1.7 compared to the wild strain. Beacham et al. [110] used a reverse protocol for *Nannochloropsis salina*, starting with exposure to EMS, followed by UV irradiation, yielding a three-fold increase in cellular lipid accumulation. Comparable results were achieved by Sivaramakrishnan and Incharoensakdi [113], who exposed *Scenedesmus* sp. to UV irradiation in combination with oxidative stress by H_2_O_2_.

Other approaches can be used to select desired microalgal cells if the results obtained by random mutagenesis are insufficient. Among them, Adaptive Laboratory Evolution (ALE) is commonly used to adapt the physiology of cells to specific process conditions, such as high temperatures [114]. Its principle is based on natural selection, as presented in the Darwinian Theory, on the laboratory bench [115], and includes extensive cultivation in a specifically designed lab environment so that enhanced phenotypes can be selected after a long period of time [116]. The environmental conditions that can be altered include light irradiation, lack of nutrients, such as nitrogen, osmotic, temperature and oxidative stress [115,117,118]. Connecting the results of ALE with whole genome sequencing and “omics” methods enables gene functions to be discovered easily [116]. However, ALE does not prevent gene instability that might occur more often than in randomly mutated cells [114,117].

Additional environmental factors can be applied on microalgae; for example, Miazek et al. [119] reviewed the use of metals, metalloids and metallic nanoparticles to enhance cell characteristics. Moreover, phytohormones or chemicals acting as metabolic precursors have already been applied to microalgae [120]. A discussion of the methods used in the latter case exceeds the scope of this review.

More recently, a new technique was developed, known as Space Mutation Breeding (SMB). This technique may have direct or indirect effects on the growth and metabolic activities of microalgae, due to the unusual environment of space, characterized by high-energy ionic radiation, space’s magnetic field, ultra-high vacuum and microgravity [121]. The SMB technique provides some advantages, such as the great improvement in species’ qualities in a short time [122]. This was achieved by Chen Zishuo et al. [121], with a seawater *Arthrospira platensis* mutant, yielding a sugar content 62.26% higher than the wild type.

## 4. Overview of High-Throughput Screening Methods and Techniques for Strain Selection

After performing random mutagenesis and providing the above cultivation conditions, mutants are analyzed and sorted to detect cells with the desired phenotypic alterations. Two main approaches can be applied, based on either quantity or quality.

### 4.1. Screening Approaches on a Quantitative Basis

The principle of these approaches is based on conducting a high number of parallel experiments, such as agar streaking or shake flasks, which are traditional methods requiring large, time-consuming and polluting equipment [123,124,125,126,127]. Process control options are, moreover, limited in these systems [22]. Microtiter plates (MTPs) have emerged and become the most widely used laboratory equipment for high-throughput screening [128,129,130,131,132,133,134,135]. Automation using laboratory robotic platforms is still required to handle the high number of parallelized processes, consisting of incubation, sample transfer, harvesting and analysis, on a reasonable time scale [131,132,135,136,137,138]. However, improvements are needed, especially with regard to robotic dispensing inaccuracy [139] and the high costs of these platforms that make them inaccessible [140].

To address these constraints, a novel cultivation strategy was recently developed, called High-Density Cultivation Screening Platform. This allows phototrophic microorganisms to be cultivated with configurations, enabling parallel cultivation, rapid growth and rapid turbulent mixing under identical conditions using a growth control unit (CellDEG GmbH, Berlin, Germany) controlling CO_2_ supply and the light profiles [141,142].

### 4.2. Screening Approaches on a Qualitative Basis

Approaches of this type are based on mutant analysis, searching for a certain characteristic at the single-cell level. This approach has to be fast, simple and cost-efficient, since the occurrence of a beneficial mutation can be very low (<1/10^5^) and as many mutated cells as possible must be analyzed and sorted [104,136,143].

Flow cytometry (FC) combined with cell sorting is one of the preferred single-cell analysis methods for high-throughput screening (HTS) [136,144]. It includes technologies that can automatically count cells, analyze their vitality, size and granularity, and identify multiple physiological states and enzyme activity with a speed reaching thousands of events per second, based on quantified scattered, fluorescent light signals [136,145]. This analysis method can be utilized to isolate and sort desired overproducing mutants [146,147], especially when combined with specific staining dyes, such as Nile Red [148,149,150] and BIODIPY [149,151], which are commonly used for intracellular lipid detection to isolate lipid-rich microalgae strains. Despite its numerous advantages, one of its main drawbacks is that extracellular target products cannot be analyzed easily, as their fluorescence signals are not associated with the cells [141,152]. The equipment’s price is high [126,153] and mechanical pressure in the sorting procedure can lead to cell disruption and not all microalgal strains survive [126,154,155].

A second HTS technique uses droplet-based microfluidic chips (also known as “lab on a chip” [136]) for single-cell level analysis, by precisely modifying the cells and their microenvironment by encapsulating each single cell in a water–oil–emulsion droplet, which creates an independent femto-, pico- or nano-liter volume bioreactor [154,156]. Thousands of uniformly fine microdroplets can be generated per second and be transported, analyzed and merged with each other, enabling high-throughput parallel processing, e.g., for screening applications [157,158] and long-term real-time monitoring [24,149,159]. Furthermore, this technology facilitates constant environmental conditions [22,159] and a high recovery rate after sorting [154], and the setup is easy to handle and can be made available relatively cheaply [151,154]. However, one serious drawback of this method is the far lower encapsulating speed to obtain microdroplets (10^3^–10^4^/s) compared to FC combined with cell sorting [136]. Furthermore, the microfluidic devices need to be specified and optimized for each experiment as unique process flows are required for every application [139].

## 5. Conclusions

Due to their multiple metabolites of interest, microalgae and cyanobacteria are promising cellular factories for biobased product synthesis. However, molecular toolboxes are not yet widely established for microalgae or the utilization of genetically modified organisms is limited by the value chain industries, such as the food industry. This aspect is the motivation to deal with approaches, which allow a natural optimization of microalgal cell factories. There is a great variety of approved physical and chemical mutagens suitable for random mutagenesis. Not all of them have been studied for microalga yet. So far, physical mutagens have been successfully applied to increase the cellular lipid or carbohydrate content of microalgae, whereas pigment production was mainly triggered by chemical mutagens, such as EMS and MNNG.

As a recent trend in scientific studies, the usage of combined mutagenesis approaches in order to increase the mutation rate of cells was identified. Nevertheless, more in-depth investigations are necessary to identify advantages and disadvantages of the different mutagenesis strategies.

Besides the mutation approaches, a co-development of high-throughput screening technologies must take place as newly generated pheno- and genotypes have to be identified and characterized regarding their new cellular functions. Additionally, there is still a need for new designs of parallelizable scale-down phototrophic cultivation systems.

## Data Availability

Not applicable.
